# Exploring the link among eating behaviour, diet quality, and relative energy deficiency in sports risk in elite Canadian volleyball male athletes

**DOI:** 10.1017/jns.2025.10046

**Published:** 2025-10-24

**Authors:** Erik Sesbreno, Louise Capling, Margo Mountjoy, Anne-Sophie Brazeau

**Affiliations:** 1 Institut National du Sport du Québec, Montréal, QC, Canada; 2 French-speaking Olympic Sports Medicine Research Network, Paris, France; 3 School of Human Nutrition, McGill University, Montréal, QC, Canada; 4 Sydney School of Health Sciences, Faculty of Medicine and Health, University of Sydney, Sydney, NSW, Australia; 5 International Olympic Committee – Games Group, Lausanne, Switzerland; 6 Department of Family Medicine, Michael G. DeGroote School of Medicine, McMaster University, Hamilton, ON, Canada

**Keywords:** Athlete Diet Index, Eating behaviour, Elite athletes, Low energy availability, Relative energy deficiency in sports, REDs, Relative Energy Deficiency in Sports, ADI, Athlete diet index, LEA, Low energy availability, TES, Testosterone, DXA, Dual Energy X-ray Absorptiometry, IOC, International Olympic Committee, ft^3^, Free-triiodothyronine, AP Spine, Anterior-Posterior lumbar spine, BMD, Bone mineral density, BMI, Body mass index, CV, Coefficient of variation, COVID, Coronavirus disease

## Abstract

Male volleyball athletes may be at risk of inadequate energy and carbohydrate intake. This may increase their risk of relative energy deficiency in sport (REDs) and impair a variety of physiological and psychological systems involved with performance and health. This study explored the eating behaviours and diet quality of international elite volleyball male athletes and their association on hormones associated with acute energy deficit and primary serum REDs indicators outlined in the International Olympic Committee REDs Clinical Assessment Tool 2. Methods: Using a retrospective design, 30 male athletes from a national indoor volleyball programme were assessed using DXA bone mineral density, hematological analysis, anthropometry, restrained eating behaviour via the Three-Factor Eating Questionnaire-R18 and the Athlete Diet Index (ADI) questionnaire. Results: All participants met or exceeded dietary recommendations for health and sport with ADI mean score of 95.2/125 ± 10.5. Restraint eating was inversely associated with insulin (*r* = − 0.37; *p*  < 0.05). Both the ADI total and core nutrition sub-scores were inversely associated with free-triiodothyronine (*r* = − 0.58; *p* < 0.01) but not with total testosterone, insulin or leptin. Conclusion: Male volleyball athletes at risk of inadequate energy intake may not necessarily demonstrate signs of poor diet quality.

## Introduction

Eating behaviour is a broad outcome resulting from the act of consumption. The set of acts include eating tendencies that developed over time, eating occasions that may be influenced by physical and/or social context, selective eating, food portions, eating disorder symptoms, and restraint eating/dieting.^(1)^ When the Three-Factor Eating Questionnaire (TFEQ)-R18 was previously used to describe the degree of restraint eating in international elite volleyball male athletes, those with low and high eating restraint habits were at risk of energy deficiency primarily from eating energy and carbohydrate below daily recommendations for moderate to high training programmes.^(2,3)^ Health and sport organisations have described multiple factors linked to the risk of inadequate energy intake in athletes such as eating disorder/disorder eating, misguided attempts to lose body mass/fat, and/or lack of skill, knowledge, or time to meet increased energy requirements for training.^(4)^

The male athlete triad model describes an inter-relationship with energy deficit, functional hypothalamic hypogonadism, and bone density.^(5)^ The consequence of inadequate energy intake was expanded to other physiological systems involved with health and sport performance in the relative energy deficiency in sports (REDs) model.^(6)^ The aetiological term that describes the condition of inadequate energy intake in both syndrome models is low energy availability (LEA).^(5,6)^ A field investigation involving international elite male cyclists revealed that higher eating restraint with the TFEQ-R18 was associated with suppressed resting metabolic rate secondary to energy deficiency.^(7)^ Reduced or low resting metabolic rate is an emerging indicator of REDs.^(6)^

The Athlete Diet Index (ADI) questionnaire was recently validated in a large cohort of national-international-world class (tier 3–5) elite athletes across multiple sport disciplines.^(8,9)^ Through a retrospective 7-day food frequency design, this online questionnaire facilitates the collection of information on eating behaviour (food group portions/day). It also facilitates the assessment of diet quality against the Australian National Healthy Eating guidelines, sport nutrition recommendations on targeted nutrients that are perceived to increase for rigorous training programmes, and desirable eating patterns near training sessions by sport nutrition experts. When assessed in female athletes, the ADI score did not associate with the risk of low energy available through the Low Energy Availability in Females Questionnaire.^(8)^ To date, there is no information exploring the relationship between eating behaviour, diet quality, and indicators of REDs in elite male athletes. Therefore, our primary aim was to describe the cognitive restraint eating behaviour, daily intake of carbohydrate-rich food serve, diet quality, and nutrient status of targeted micronutrients/minerals of elite male volleyball athletes. Our secondary aim was to explore the relationship between eating behaviour, diet quality, physique traits, and serum hormones influenced by energy deficit.

## Methods

### Participants

Tier 4 international elite male volleyball players (*n* = 30, ≥ 18 years old) were recruited through verbal invitation by the primary investigator near the end (August) of the national team season in 2021.^(9)^ Players were training at the national training centre in Gatineau, Quebec, Canada, in preparation for international competition. The inclusion criteria were players invited to the national training centre to train in preparation for in-season international competition for team Canada and completed week-1 baseline testing in the TFEQ-R18 and ADI questionnaires, anthropometry, bone mineral density (BMD) scans with dual energy x-ray absorptiometry (DXA), and fasted morning bloodwork. Those inactive due to injury on week 1 of training camp were excluded. Athletes provided written informed consent. The study was approved by the McGill University’s Research Ethics Board 4 in accordance with the declaration of Helsinki (21-09-035).

### Design

A retrospective cross-sectional analysis was performed on aggregated data that was collected on week 1 during a 5-week team training camp between May and August 2021. All tests, except for BMD, were administered at the beginning of week-1 between 8 and 10 AM in a fasted state before exercise. BMD was assessed between 9 and 12 PM during week 1 of training camp in a non-fasted state. At the time of the assessment, the team training schedule involved three volleyball on-court competitive play/scrimmage (metabolic equivalent of task (MET) 6.0) for ∼90–120 min per session, three on-court skill building exercises such as serves, serve, and pass (MET 3.0) for ∼60–90 min per session, and three strength training sessions (MET 6.0) for ∼60 min per session during the week.^(10)^ Training loads and methods prior to camp were self-directed and lacked sufficient detail for an accurate description.

### Eating behaviour and diet quality

The TFEQ-R18 refers to current dietary practice and measures three different aspects of eating behaviour: restrained eating (conscious restriction of food intake to control body weight or to promote weight loss), uncontrolled eating (tendency to eat more than usual due to a loss of control over intake accompanied by subjective feelings of hunger), and emotional eating (inability to resist emotional cues).^(2)^ The questionnaire consists of 18 items on a 4-point response scale and items were scored by hand and totalled for a final overall score. To focus the investigation on cognitive restraint practices, only the cognitive restraint (6 questions out of 18) was assessed. A high relative restraint eating score was defined as a value within the upper tertile of the group’s score. de Lauzon et al. reported that that with a cross-sectional sample of young adults (non-athletic) (*n* = 500+), those with a cognitive restraint eating score within the upper tertile of the group’s score demonstrated a predominantly higher consumption of healthier foods and female participants demonstrated a lower energy intake.^(2)^

Frequency and type of different foods and fluids consumed over the last 7 days was assessed with the online ADI with automated scoring.^(8)^ It is a novel diet quality assessment tool for sport nutrition experts to efficiently complete dietary assessments with athletes in the field. The ADI was created with the assistance of expert clinicians in sport nutrition and validated in elite athletes. The total ADI score, sub-scores (i.e. core nutrition, special nutrients, and dietary habits), and non-scored information (i.e. 7-day training log, special diets, and dietary supplement use) were used in combination to provide an indication of the overall diet quality and dietary patterns of athletes. The core sub-score involves the reporting of serves of food associated with healthy eating (i.e. fruit, vegetables, grains, breads and cereals, dairy products and alternatives and meat and alternatives as well as discretionary foods and alcohol); indicators for specific micronutrients such as calcium and iron that might have increased requirements in athletes and patterns of dietary behaviour specific to athletes undertaking an intensive training schedule. A maximum quality score (out of a possible 125) was applied for participants who met recommendations, with pro-rated scores applied for lower intakes and less desirable habits (e.g. skipping one or more main meals on a regular basis). A score ≥ 90 was classified as exceeding recommendations; a score 66–89 was classified as meeting recommendations, while a score ≤ 65 was classified as below recommendations. Non-scored data were collected such as medical conditions, injuries, weight goals, as descriptive data to help contextualise the dietary trends and scores. Discretionary (i.e. candy, potato chips, cookies, etc.) and nondiscretionary (i.e. fruit, starchy vegetables, dairy, and cereal/grains) servings were described to inform on eating behaviour of carbohydrate-rich foods due to the risk of inadequate carbohydrate intake in volleyball players and its association on poor energy intake.^(3)^ Because these eating behaviours are best reflected in the ADI core sub-score, the total ADI and core sub-scores will be emphasised for group analysis. The ADI questionnaire does not quantify the daily use of dietary supplements and sports foods; thus, these potential sources of carbohydrates will be excluded from the estimates of daily intake of total carbohydrate-rich foods serve.

### Blood samples

All participants provided morning blood samples after a minimum 10-hour fast. Serum was separated by centrifugation (3000–3500rpm for 10–15 minutes) and then analysed for leptin (enzyme immunoassay) and insulin (enzyme immunoassay) given their potential sensitivity to acute energy deficiency conditions,^(11)^ free-triiodothyronine (ft^3^) (chemiluminescent immunoassay), free- (calculated from total-testosterone (TES), sex hormone binding globulin and albumin), and total-TES (liquid chromatography-tandem mass spectrometry) given they are primary indicators of REDs,^(6)^ ferritin (enzyme immunoassay), and red blood cell (RBC) magnesium (unavailable when requested). Serum was analysed by an accredited laboratory (Lifelabs Medical Laboratories, Ottawa, Ontario, Canada). The sampling and storage protocols were performed as previously described.^(3)^ Analytic coefficient of variation (CV)%: fasted insulin (3.01%), total TES (1.57%), fasted glucose (1.02%), ft^3^ (2.04%), and free-TES (calculated by the commercial laboratory). Analytic CV% was not available for leptin, ferritin, and RBC magnesium.

### Anthropometry

Body mass, standing height, and skinfolds at eight sites were measured at the training centre on week 1 by the same level III accredited anthropometrist from the International Society for the Advancement of Kinanthropometry (ISAK) using techniques and procedure outlined in the ISAK manual.^(11)^ Standing height was measured using a stadiometer (Rosscraft, Surrey, BC, Canada), body mass on a calibrated digital scale with a precision of ± 0.1 kg (BWB-800S Tanita, Illinois, USA), and skinfolds with a Harpenden calipers (Baty International, Burgess Hill, England). The equation used to calculate body mass index (BMI) was body mass (kg)/standing height (cm).

### Bone mineral density

Anterior-posterior lumbar spine (AP Spine) and femur BMD were assessed in a non-fasted state via dual-energy x-ray absorptiometry (Lunar Prodigy, GE Healthcare, Madison, WI, USA) according to standard positioning protocol from the International Society for Clinical Densitometry.^(13)^ The USA (Combined NHANES (ages 2030)/Lunar (ages 20–40)) Total Body Reference Population (v113) was used as the reference database with analysis performed using GE Encore version 13.60 software (GE, Madison, WI, USA).

### Signs of relative energy deficiency in sports

Participants at potential risk of REDs were characterised by using available primary indicators such as total-TES (< 13.5nmol/L = < 25 percentile of normal range), free-TES (< 306pmol/L = < 25 percentile of normal range), ft^3^ (< 3.5pmol/L= < 25 percentile of normal range), and spine or femur BMD (< − 1.0 z score) indicators in accordance with the International Olympic Committee (IOC) REDs Clinical Assessment Tool (CAT) 2.^(14)^ The primary investigator completed the REDs scoring and reviewed the medical history that was submitted by each player during week 1 of the training camp as part of the medical screen. No diagnosis was found that may impact REDs biomarkers.^(14)^

### Statistics

Statistical analyses were carried out using SPSS (SPSS v23.0; IBM Inc, Chicago, IL, USA). In order to detect a group mean difference (score of 14, *β* = 0.8; *α* = 0.05) in ADI total score, a sample size of 26 participants was required.^(8)^ Descriptive statistics are presented as mean ± standard deviation. Normality was assessed via the Shapiro–Wilk test. Pearson’s (normally distributed data) or Spearman’s (non-normally distributed data) correlation coefficient was used to assess the relationship among cognitive restraint eating score, total carbohydrate intake serve per day, ADI total and core nutrition sub-scores, total sum of eight skinfolds, BMI, total-TES, insulin, ft^3^, and leptin. Independent variables were TFEQ-R18 — cognitive restraint eating score (upper tertile vs rest) and REDs risk status (at-risk vs no/minimal risk) and all remaining items were dependent variables. Mean differences between groups were analysed by independent t-test (normally distributed data) or Mann–Whitney U (non-normal distributed data). Significance was set to *p* < 0.05, and a Bonferroni-adjusted alpha level *p* < 0.01 was applied for repeated testing. Hedges’ correction effect size based on 95% confidence interval was calculated and interpreted as < 0.2 (trivial), 0.2–0.5 (small), 0.5–0.8 (moderate), and > 0.8 (large).^(15)^ The effect with CI crossing the upper and lower boundaries of ±0.3 was described as unclear.^(16)^

## Results

Characteristic of eating behaviour, diet quality, and supplement use of all 29 athletes recruited from the Canadian men’s national indoor volleyball programme are shown in Table 1. Nearly 50% of participants trained over 20 hours per week prior to training camp (data not shown). Athletes had a total ADI score (range = 72–111) that meet or exceeding recommendations (≥ 66). Participants, on average, consumed ∼8 serving/d of fruit and vegetables, ∼6 serving/d of grain/cereals, ∼3 serving/d of dairy, ∼2 serving/d meat/alternative, and ∼3 serving/week of alcoholic beverages. Seventy per cent (*n* = 21) of participants reported using at least one dietary supplementation with ≥ 50% of participants taking protein and sports foods (gels/bars). Based on normal laboratory ranges, three athletes had low leptin level (< 1.8ng/ml BMI > 25.0kg/m^2^), two had low insulin level (< 20pmol/L), and five had low magnesium level (< 1.65mmol/L). Three athletes had ferritin levels < 35ug/L and one athlete had a borderline low BMD (− 1.0 z score).

**Table 1.** Eating behaviours and diet quality

Eating behaviour	All (*n* = 29)
Cognitive restraint eating score	12.1 ± 3.4 (7–20)
Fruits (serving/day)	4.2 ± 1.6 (2–8)
Starchy vegetables (serving/day)	1.6 ± 1.2 (0–5)
Non-starchy vegetables (serving/day)	3.0 ± 1.4 (1–5)
Grains/cereals (serving/day)	6.2 ± 2.6 (3–12)
Dairy (serving/day)	3.1 ± 1.3 (1–5)
Meat and alternatives (serving/day)	2.4 ± 0.6 (1–3)
Alcohol (serving/week)	3.7 ± 3.1 (0–8)
*Carbohydrate-rich foods*	
Non-discretionary food sources (serving/day)	14.1 ± 3.9 (9–24)
Discretionary food sources (serving/day)	1.1 ± 0.7 (0–2.3)
Combined foods sources (serving/day)	15.2 ± 3.9 (9–26)
**Diet quality**	
Athlete Diet Index total score (max 125)	95.2 ± 10.5 (72–111)
Core nutrition sub-score (max 80)	61.1 ± 9.0 (41–75)
Special nutrients sub-score (max 35)	25.1 ± 3.2 (16–32)
Dietary habits sub-score (max 10)	9.0 ± 0.9 (8–10)
**Dietary supplement reportedly used in the past 4 weeks**	
Probiotics	1 (3)
Omega 3	5 (17)
Electrolyte	6 (23)
Antioxidants	6 (23)
Caffeine	8 (27)
Multivitamin	11 (37)
Creatine	11 (37)
Sport drink	13 (43)
Calcium	14 (47)
Iron	14 (47)
Sports foods (gel or bar)	15 (50)
Protein	29 (97)

Data presented as mean ± standard deviation (range) or n (%).

Table 2 describes differences in eating behaviour, diet quality, physique traits, bloodwork, bone density, training history, and REDs risk status between high restraint eating score participants versus the rest of participants. The group with high restraint eating behaviour had a TFEQ-R18 value between 13 and 20, whereas the rest of participants ranged from 7 to 12. The higher restraint group were older and had lower calcidiol values than the rest of participants. There was a small meaningful effects size difference to indicate that the higher restraint eating group had a higher ADI total and core sub-scores, higher skinfold value, higher BMI, higher lumbar spine and femur BMD, higher leptin, and lower RBC magnesium values. Three athletes with mild signs of REDs where categorise as higher restraint eatings and two athletes with mild signs of REDs were in the other group.

**Table 2.** Characteristics of athletes with high restraint eating behaviour

	High restraint eating (*n* = 11)	Rest (*n* = 18)	*p* value; effect size (95 % CI)
Age (years)	22.3 ± 2.3 (18–25)	20.5 ± 1.2 (18–23)	**0.01;** − **0.9 (**− **1.7–(**− **0.18))**
**Eating behaviours and diet quality**			
TFEQ-R18 score	15.5 ± 2.4 (13–20)	9.9 ± 1.6 (7–12)	**0.001;** − **2.7 (**− **3.8–(**− **1.7))**
Carbohydrate foods (serve/day)	15.4 ± 3.3 (12–21)	15.1 ± 4.3 (9–26)	0.88; 0.0 (− 0.7–0.6)
ADI core sub-score	64.5 ± 7.3 (48–75)	58.9 ± 9.7 (41–75)	0.11; − **0.6 (**− **1.3–0.1)**
ADI total score	98.3 ± 9.2 (81–109)	93.3 ± 11.2 (72–111)	0.20; − **0.4 (**− **1.2–0.2)**
**Anthropometry**			
Standing height (cm)	195.6 ± 8.7 (175.7–203.0)	196.0 ± 7.8 (170.0–207.4)	0.46; 0.0 (− 0.6–0.7)
Body mass (kg)	92.2 ± 7.4 (72.897.0)	89.6 ± 7.9 (72.1–102.5)	0.29; − 0.3 (− 1.0–0.4)
Sum of eight skinfolds (mm)	71.2 ± 10.4 (52.6–86.1)	62.3 ± 14.1 (40.6–88.4)	0.08; − **0.6 (**− **1.4–0.0)**
Body fat (%)			
Body mass index (kg/m^2^)	24.0 ± 1.2 (21.7–27.0)	23.3 ± 1.5 (21.0–26.0)	0.19; − **0.4 (**− **1.2–0.2)**
Lean mass index (mm·kg^−^ ^0.14^)	51.9 ± 4.7 (40.0–56.3)	51.2 ± 3.9 (43.2–57.8)	0.23; − 0.1 (− 0.9–0.5)
**Reproductive hormones**			
Total testosterone (nmol/L)	19.1 ± 5.4 (10.9–29.8)	18.9 ± 3.8 (11.0–27.5)	0.92; 0.0 (− 0.7–0.6)
Free testosterone (pmol/L)	398.5 ± 116.6 (240.0–607.0)	416.7 ± 88.0 (253.0–596.0)	0.63; 0.1 (− 0.5–0.9)
**Bone density**			
Lumbar spine (g/cm^2^)	1.442 ± 0.085 (1.270–1.640)	1.378 ± 0.147 (1.100–1.630)	0.18; − **0.4 (**− **1.2–0.2)**
Femur (g/cm^2^)	1.405 ± 0.127 (1.210–1.670)	1.337 ± 0.134 (1.100–1.580)	0.19; − **0.4 (**− **1.2–0.2)**
**Metabolic hormones**			
Leptin (ng/ml)	0.8 ± 0.6 (0.3–2.4)	0.5 ± 0.2 (0.3–1.2)	0.20; − **0.6 (**− **1.3–0.1)**
ft^3^ (pmol/L)	4.8 ± 0.7 (3.4–6.2)	4.9 ± 0.6 (4.1–6.2)	0.69; 0.1 (− 0.5–0.8)
Insulin (pmol/L)	33.9 ± 21.6 (9.0–91.0)	42.7 ± 19.2 (16.0–77.0)	0.17; 0.4 (− 0.3–1.1)
Risk of relative energy deficiency in sports N (%)*			
No-minimal risk	8 (73)	16 (89)	
Mild risk	3 (27)	2 (11)	
**Nutrient status**			
Ferritin (ug/L)	85.8 ± 49.2 (23.0–195.0)	78.6 ± 36.2 (29.0–162.0)	0.52; − 0.1 (− 0.8–0.5)
Calcidiol (nmol/L)	83.0 ± 27.9 (47.0–149.0)	101.6 ± 19.3 (64.0–139.0)	**0.04; 0.7 (0.0–1.5)**
RBC magnesium (mmol/L)	1.75 ± 0.16 (1.60–2.10)	1.84 ± 0.19 (1.50–2.16)	0.16; **0.4 (**− **0.2–1.2)**
**Player positions N (%)**			
Libero	1 (9)	1 (5)	
Setters	2 (18)	3 (17)	
Hitters/blockers	8 (73)	14 (78)	
**Training schedule N (%)**			
11–20 hours/week	7 (64)	10 (56)	
20+ hours/week	4 (36)	8 (44)	
**Nutrient status below normal limits N (%)**			
Calcidiol (< 75nmol/L)	5 (45)	2 (11)	
RBC magnesium (< 1.65 mmol/L)	3 (27)	2 (11)	

Data are presented as mean ± standard deviation (range); **(bold) significant ES difference and/or** ***p*****< 0.05**, *Based on relative energy deficiency in sports CAT2 scoring tool; TFEQ-R18, Three-Factor Eating Questionnaire-R18; RBC, red blood cell; ft^3^, free-triiodothyronine; ADI, Athlete Diet Index.

Table 3 describes differences in eating behaviour, diet quality, physique traits, bloodwork, bone density, and training history between participants at mild risk of REDs (*n* = 5 (17%)) versus those at no-minimal risk (*n* = 24 (83%)). In terms of primary serum REDs indicators, there were three cases of low subclinical total-TES, five cases of low subclinical free-TES, and one borderline low subclinical ft^3^ (< 3.5pmol/L). Those at mild risk of REDs had lower TES values and higher femur BMD values. There was a small meaningful effects size difference to indicate that the group at mild risk of REDs had a higher ADI core sub-score value and a lower RBC magnesium value compared to the no-minimal risk group.

**Table 3.** Characteristics of athletes at mild risk of relative energy deficiency in sports

	Mild risk REDs status (*n* = 5)	No-minimal risk REDs status (*n* = 24)	*p* value; Effect size (95 % CI)
Age (years)	21.7 ± 2.5 (20–26)	21.1 ± 1.8 (18–26)	0.58; − 0.2 (− 1.2–0.6)
**Eating behaviours and diet quality**			
TFEQ-R18 score	13.6 ± 4.9 (7–19)	11.7 ± 2.9 (8–20)	0.40; − 0.5 (− 1.4–0.4)
High restraint eating, n (%)	6 (60%)	8 (33%)	
Carbohydrate foods (serve/day)	14.6 ± 2.3 (12–17)	15.3 ± 4.2 (9–26)	0.27; 0.1 (− 0.7–1.1)
ADI Core sub-score	66.6 ± 6.8 (55–71)	59.9 ± 9.3 (41–75)	0.11; − **0.7 (**− **1.6–0.2)**
ADI Total score	100.6 ± 9.0 (86–109)	93.9 ± 10.7 (72–111)	0.21; − 0.6 (− 1.5–0.3)
**Anthropometry**			
Standing height (cm)	197.1 ± 9.2 (184.3–207.4)	195.6 ± 7.9 (170.0–205.9)	0.60; − 0.1 (− 1.1–0.7)
Body mass (kg)	91.9 ± 5.4 (85.1–99.2)	90.3 ± 8.1 (72.1–102.5)	0.90; − 0.1 (− 1.1–0.7)
Sum of skinfolds (mm)	71.8 ± 15.3 (49.8–88.4)	64.4 ± 12.9 (40.6–86.1)	0.27; − 0.5 (− 1.4–0.4)
Body mass index (kg/m^2^)	23.7 ± 1.3 (21.7–25.1)	23.5 ± 1.5 (21.0–27.0)	0.86; 0.0 (− 1.0–0.8)
Lean mass index (mm·kg^− 0.14^)	51.7 ± 3.2 (47.1–54.7)	51.4 ± 4.4 (40.0–57.8)	0.90; 0.0 (− 0.9–0.8)
**Reproductive hormones**			
Total testosterone (nmol/L)	12.3 ± 1.8 (10.9–14.5)	20.3 ± 3.9 (14.6–29.8)	**0.001; 2.0 (0.9–3.0)**
Free testosterone (pmol/L)	270.4 ± 22.5 (240.0–291.0)	438.8 ± 80.8 (342.0–607.0)	**0.001; 2.1 (1.0–3.2)**
**Bone density**			
Femur (g/cm^2^)	1.469 ± 0.123 (1.350–1.670)	1.341 ± 0.126 (1.100–1.580)	**0.04;** − **0.9 (**− **1.9–0.0)**
Lumbar spine (g/cm^2^)	1.426 ± 0.010 (1.410–1.440)	1.397 ± 0.142 (1.100–1.640)	0.33; − 0.2 (− 1.1–0.7)
**Metabolic Hormones**			
Leptin (ng/ml)	0.5 ± 0.2 (0.3–0.9)	0.7 ± 0.4 (0.3–2.4)	0.29; 0.4 (− 0.4–1.3)
ft^3^ (pmol/L)	4.4 ± 0.6 (3.4–5.1)	4.9 ± 0.6 (4.1–6.2)	0.15; 0.6 (− 0.3–1.5)
Insulin (pmol/L)	28.4 ± 17.3 (9.0–56.0)	41.7 ± 20.3 (16.0–91.0)	0.17; 0.4 (− 0.3–1.1)
**Nutrient Status**			
Ferritin (ug/L)	94.4 ± 31.3 (69.0–147.0)	78.6 ± 42.7 (23.0–195.0)	0.13; − 0.3 (− 1.3–0.5)
Calcidiol (nmol/L)	90.8 ± 18.1 (64.0–114.0)	95.4 ± 25.6 (47.0–149.0)	0.70; 0.1 (− 0.7–1.1)
RBC Magnesium (mmol/L)	1.66 ± 0.10 (1.50–1.77)	1.84 ± 0.19 (1.60–2.16)	0.08; **0.9 (0.0–1.9)**
**Player positions N (%)**			
Libero	0 (0)	2 (8)	
Setters	2 (40)	3 (13)	
Hitters/Blockers	3 (60)	19 (79)	
**Training Schedule N (%)**			
11–20 hours/week	4 (80)	13 (54)	
20+ hours/week	1 (20)	11 (46)	
**Nutrient Status Below Normal Limits N (%)**			
Calcidiol (< 75nmol/L)	1 (20)	6 (25)	
RBC Magnesium (< 1.65 mmol/L)	2 (66)	3 (13)	

Data presented as mean ± standard deviation (range); **(bold) significant ES difference and/or** ***p*****< 0.05**, * Based on relative energy deficiency in sports CAT2 scoring tool; TFEQ-R18, Three-Factor Eating Questionnaire-R18; RBC, red blood cell; ft^3^, free-triiodothyronine; ADI, Athlete Diet Index.

Table 4 describes the relationship among restraint eating score, combined daily portions of carbohydrate-rich foods consumed, diet quality, physique traits, primary serum indicators of REDs (TES and ft^3^), and serum hormones influenced by energy deficit in male subjects (insulin and leptin). There was a significant (*p* < 0.01) positive association between ADI total and core sub-scores. There was a significant inverse correlation with ADI total and core sub-scores and ft^3^ values. There was a significant positive correlation between ft^3^ and insulin. There was a positive correlation (*p* < 0.05) between combined carbohydrate food serve per day and ADI core sub-score as well as between ft^3^ and total TES values. There was a negative correlation (*p* < 0.05) between TFEQ-R18 restraint eating score and insulin as well as ADI total score and BMI.

**Table 4.** Spearman’s or Pearson’s correlation coefficients are reported to describe the association between restraint eating scores, diet quality scores, physique traits and hormones commonly associated with low energy availability

		1	2	3	4	5	6	7	8	9
1	Restraint eating score									
2	Combined CHO food serves	0.04 (0.82)								
3	ADI core sub-score	0.26 (0.18)	0.37* (0.04)							
4	ADI total score	0.21 (0.31)	0.28 (0.12)	**0.95** (0.001)						
5	Total skinfolds (mm)	0.24 (0.19)	− 0.18 (0.43)	− 0.28 (0.14)	− 0.35 (0.06)					
6	Body mass index (kg/m^2^)	0.18 (0.30)	0.02 (0.95)	− 0.28 (0.16)	− 0.36* (0.04)	0.13 (0.48)				
7	Total-testosterone (nmol/L)	0.00 (0.92)	0.10 (0.57)	− 0.25 (0.21)	− 0.29 (0.11)	− 0.23 (0.22)	0.20 (0.28)			
8	ft^3^ (pmol/L)	− 0.10 (0.39)	− 0.18 (0.25)	− **0.58** (0.001)	− **0.58** (0.001)	0.28 (0.10)	0.23 (0.22)	0.38* (0.04)		
9	Insulin (pmol/L)	− 0.37* (0.04)	− 0.13 (0.48)	− 0.25 (0.18)	− 0.21 (0.27)	0.17 (0.36)	− 0.17 (0.36)	0.03 (0.87)	**0.52** (0.001)	
10	Leptin (ng/ml)	0.24 (0.20)	− 0.16 (0.39)	− 0.36 (0.05)	− 0.35 (0.06)	**0.59** (0.001)	0.15 (0.46)	0.12 (0.51)	0.34 (0.06)	0.27 (0.17)

*N* = 29, significance (two-tailed) *p* ≤ 0.05*; *p* < 0.01 (bold) Bonferroni-corrected.

## Discussion

Prior to commencing a 5-week training camp, international elite volleyball players met or exceeded dietary recommendations for health and sport. Despite the high scores observed for the ADI total score and the ADI core nutrition sub-score, inverse associations between those scores and serum ft^3^ level were observed. ft^3^ is one of the primary serum REDs indicators outlined in the IOC REDs CAT2 severity/risk assessment tool.^(14)^

The quality of dietary intake observed in our sample was consistent with an earlier investigation that reported a higher total score in team compared to individual sport athletes.^(18)^ However, our findings contrast with a previous observation that athletes training fewer hours per week (< 12hrs/wk) are likely to score higher dietary habit sub-score (> 9.0) than those training longer hours because more time was presumably available for food preparation.^(17)^ Potentially, better food choices and shared culinary responsibilities among family members and/or roommates, as a consequence of municipalities restricting public mobility during the coronavirus could partially explain the score disease (COVID)-19 pandemic, may have improved dietary intake at home^(19)^ and observed. The high proportion of dietary supplement use for health and sport performance is consistent with previous observations on elite Canadian athletes.^(20)^ Nevertheless, athletes with observed iron depletion (ferritin < 35ug/L)^(21)^ and low normal RBC magnesium level may benefit from expert nutrition support to individualise changes to eating behaviour and/or targeted micronutrient supplementation to correct for risk of nutrient deficiency.

The inter-relationship among restraint eating, metabolic hormones, fat mass, and reproductive hormones appear consistent with conditions associated with energy deficit in men.^(7,22–24)^ However, the ADI core nutrition sub-score had an unexpected inverse association with the ft^3^ value. It appears that energy deficit was associated with a diet with a higher intake of carbohydrate-rich foods. Our sample was unlikely to be in chronic energy deficit since 97% of participants were above the low subclinical ft^3^ threshold based on the REDs CAT2.0 scoring matrix, and there was no association between those at mild and no-minimal risk of REDs nor those with higher restraint eating behaviour assessed by the TFEQ-R18.^(14)^ Similar to an earlier investigation on elite male volleyball players, no participants, despite some at mild risk of REDs, were at risk of low BMD.^(25)^ This could be associated with the positive effects of mechanical loading on the bone secondary to repetitive jumping.^(26)^ To our surprise, BMD was significantly higher in those at mild risk of REDs, but this could be that athletes in this group were older after accounting for the age ranges. Those with low subclinical free-TES may have been at risk of overtraining syndrome (OTS) from intentionally training hard while adopting a healthy diet to prepare for upcoming national team commitments.^(27)^ Unfortunately, the absence of sport specific performance data makes it impossible to distinguish OTS in this sample. A possible alternative explanation is that athletes with a high diet quality have been reported to have low intake of discretionary food, which may in turn, impact total energy intake.^(28)^

For sport nutrition professionals, the relative ease of accessibility to information on eating behaviour and diet quality as demonstrated by the ADI questionnaire may help to screen for potential areas for supporting athletes with health and/or sport related objectives.^(8,18)^ Our findings also indicate that volleyball athletes who demonstrate good indices of dietary intake through the ADI scoring system may still be at risk of insufficient energy intake while training based on the results from the REDs CAT2 scoring system. Therefore, the inclusion of primary and secondary REDs indicators to detect signs of problematic energy deficit along with performance change outcomes to exclude the risk of OTS may be useful to compliment the assessment of eating behaviours and dietary intake in free-living athletes to focus dietary support interventions on LEA as needed.

### Limitations

Several decisions around study design may have impacted our assessment. First, despite the potential of the ADI questionnaire to enhance accessibility of information on eating behaviour and diet quality of elite athletes, the specific analysis of dietary intake (i.e. total energy and macronutrient intake) and energy exercise expenditures were not assessed for a direct assessment of energy availability to inform risk of LEA. However, the attempt to explore the relationship among eating behaviour, diet quality, and LEA risk was important to inform nutrition professional of the potential utility of the ADI questionnaire to manage athletes at risk of LEA. Second, the eating behaviours that characterise our sample were potentially influenced by the COVID-19 pandemic and, thus, may limit our ability to generalise our findings to the post-COVID period. Third, the point-prevalence cross-sectional design does limit our ability to link eating behaviours and diet quality to time-dependent clinical outcomes as we were able to identify mild cases of REDs, cases of nutrient inadequacy, or deficiency despite participants demonstrating good dietary habits for health and sport. Fourth, training loads prior to camps likely affected the baseline results. The lack of accurate information to describe self-directed training prior to camp is an important limitation given that it could impact LEA risk. Finally, the core nutrition sub-score was based on the Australian Dietary Guidelines. However, the principles underpinning the guidelines were reflected in the Canadian Food Guide.^(29,30)^

## Conclusion

Male volleyball athletes assessed in this investigation met or exceeded recommendations for health and sport. To our surprise, dietary quality measured by the ADI questionnaire was inversely related to ft^3^ which contrasts our initial hypothesis. We cannot demonstrate that diet quality as assessed by ADI questionnaire is associated with the risk of REDs in these athletes. Future work should explore eating behaviours and diet quality in volleyball players during the post-COVID period.
